# Humanized scFv Molecule Specific to an Extracellular Epitope of P2X4R as Therapy for Chronic Pain Management

**DOI:** 10.3390/cells14130953

**Published:** 2025-06-22

**Authors:** Adinarayana Kunamneni, Karin N. Westlund

**Affiliations:** 1 Department of Internal Medicine, Mayo Clinic, Jacksonville, FL 32224, USA; kunamneni.adinarayana@mayo.edu; 2 Department of Anesthesiology and Critical Care Medicine, University of New Mexico Health Sciences Center, Albuquerque, NM 87101, USA

**Keywords:** neuropathic pain, nerve injury, single-chain Fragment variable (scFv), ribosome display, trigeminal nerve, sciatic nerve, cytokine, chronic pain, animal model, allodynia, non-opioid

## Abstract

Chronic pain affects a significant portion of the population, with fewer than 30% achieving adequate relief from existing treatments. This study describes the humanization methodology and characterization of an effective non-opioid single-chain fragment variable (scFv) biologic that reverses pain-related behaviors, in this case by targeting P2X4. After nerve injury, ATP release activates/upregulates P2X4 receptors (P2X4R) sequestered in late endosomes, triggering a cascade of chronic pain-related events. Nine humanized scFv (hscFv) variants targeting a specific extracellular 13-amino-acid peptide fragment of human P2X4R were generated via CDR grafting. ELISA analysis revealed nanomolar binding affinities, with most humanized molecules exhibiting comparable or superior affinity compared to the original murine antibody. Octet measurements confirmed that the lead, HC3-LC3, exhibited nanomolar binding kinetics (KD = 2.5 × 10^−9^ M). In vivo functional validation with P2X4R hscFv reversed nerve injury-induced chronic pain-related behaviors with a single dose (0.4 mg/kg, intraperitoneal) within two weeks. The return to naïve baseline remained durably reduced > 100 days. In independent confirmation, the spared nerve injury (SNI) model was similarly reduced. This constitutes an original method whereby durable reversals of chronic nerve injury pain, anxiety and depression measures are accomplished.

## 1. Introduction

Of Americans with chronic pain, 30% suffer from chronic back/sciatica pain and over 23% of these suffer from head/neck pain. Less than 30% are effectively treated by current therapies [1]. Injury-induced ATP activates P2X4, a non-specific channel member of the purinergic family well known for signaling a cascade of events promoting pain and inflammation involving BDNF and TNF alpha. Described here is the methodology used to generate humanized P2X4R scFvs (hscFv) that bind human, rat, and mouse targets. A panel of nine humanized small single-chain variable fragment (hscFv) antibodies was generated from a lead murine parent mscFv we described previously [2]. The resulting humanized scFv had a binding affinity in the nanomolar range, 100-fold better than that of the murine parent mscFv we reported previously [2]. The humanized hscFv also recognized the target intended in the extracellular portion of the human P2X4 receptor which was a 13-amino-acid peptide fragment with no sequence homology with other purinergic receptors in database searches. The targeted extracellular peptide sequence is not the ATP activation site.

The hscFv was generated with cell-free ribosome display technology and recombinant antibody selection applied as described here. We performed pilot in vivo validation with a surgically induced model of trigeminal nerve injury mimicking the human neuropathic pain condition [2]. It is not uncommon that humanization will restrict continued binding to murine sequences. However, the hscFv lead selected instead provided distinct improved efficacy in behavioral testing. The lead reversed pain related behaviors to baseline. The humanized P2X4R hscFv, by reversing the neuropathic pain, prevented the anxiety- and depression-like behavioral measures that developed in the untreated mice in weeks 6–8. Ribosome display technology described here is a novel means for generating highly effective scFv with durable therapeutic potential and no noted side effects.

## 2. Materials and Methods

### 2.1. Target Sequence

The sequence of the target extracellular P2X4 receptor peptide fragment selected corresponds to amino acid residues 301–313 (C-RDLAGKEQRTLTK, MW 1516 g/mol) in rat, with 11/13 amino acid residues identical to the human sequence. This is not the ATP binding site that activates P2X4. Binding of murine mscFv95 for the rat P2X4R peptide was estimated to be 130–190 nM in our previous study [2].

### 2.2. P2X4R scFv Humanization and Upscaling Overview

**Figure 1:**
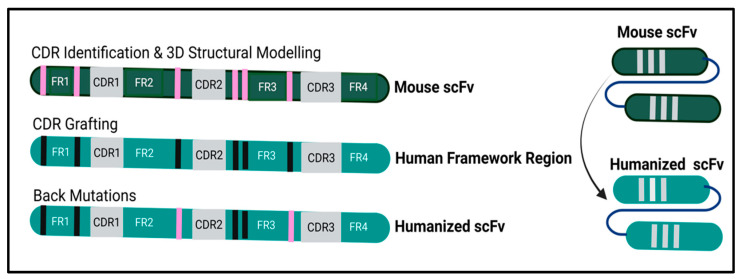
Humanization/CDR grafting technology. For the humanization/CDR grafting process, the murine amino acid sequence was loaded into BioLuminate (Schrodinger release 2022-3), with annotations made for the regions of FRs and CDRs. The 3D structure model of the antibody was constructed based on FR and CDR templates. The best mode was selected based on sequence similarity and energy minimization. BioLuminate identified three types of canonical structure-determining residues. Using 3D visualization of the structure, the roles of the residues in maintaining conformation were confirmed. The murine CDRs were grafted onto the selected human FRs with the highest homology in the scFv sequence database. Finally, key residues were designed for back-mutation. This was based on the previously identified canonical structure determining residues and predictions of their effects on binding ability and structure maintenance.

The amino acid sequence P2X4R mouse mscFv95 was loaded into the Schrödinger’s BioLuminate 5.8 software and FRs or CDRs were identified by the Kabat numbering scheme (Figure 1). The precise structure of mscFv95 was built using this software. The model was used to identify homology with known human variable heavy (VH) and light (VL) sequences using BioLuminate. Three genes with the most homology were identified that maintained the following key criteria: framework homology, key framework residues, and canonical loop structure. This analysis involved a combined IMGT/Kabat CDR labelling approach using the BioLuminate software (Schrodinger).

The three humanized VH domains and three humanized VL domains were synthesized. After cloning into the pET32 expression vector, the humanized scFvs were expressed in Rosetta-gami competent cells. Nine humanized scFv variants resulted that were used for recombinant production, purification, and affinity measurement. The candidate scFvs selected were evaluated for productivity and yield after a His Tag affinity purification. Kinetic analysis was performed to compare the human and mouse peptides.

The variants expressed in the bacterial cytoplasmic extracts (CPEs) were tested to assess affinity for the human and the rat target P2X4R peptides. The binding to the target peptides, determined during the humanization process, involved using biotinylated peptides immobilized as ligands to test the humanized scFv as analytes. The humanized hscFv versions bound equally well to the rat and human peptides. The custom peptides used in the characterization assays were synthesized by GenScript.

Resultant recombinant chimeric humanized scFvs were purified and kinetic interaction assessed by both ELISA and Octet. The lead clone had an equilibrium dissociation constant (KD) of < 130 nM. The lead clone was further assayed for purity and endotoxin requirements.

### 2.3. Sequencing the Humanized hscFv

For the construct of humanized hscFv variants, DNA was amplified with the forward primer RDT7-5′CTATAGAAGGGTAATACGACTCACTATAGGGCGAATTCC**ACCATGG**CC3′ (with an NcoI restriction sequence underlined and highlighted in bold) and the reverse primer MVLR-5′AGT**GCGGCCGC**ATCAGCCCGTTTTATTTCCAA3′ (with an NotI restriction sequence underlined and highlighted in bold) [3,4,5]. Both sets of primers allowed subcloning of the two amplicons into a pET32a-His vector. The plasmids were transformed into Rosetta-gami (DE3) *E. coli* strain and plated onto LB agar (carbenicillin 100 µg/mL, 37 °C, 16 h). Isoelectric points and molecular weight were predicted using the ExPASy bioinformatics resource portal [6]. The next day, five colonies were inoculated into LB media (3 mL, carbenicillin (100 µg/mL), 37 °C) with 225 rpm shaking (16–18 h). Positive clones were selected by restriction analysis (NcoI, NotI) and their identity confirmed by DNA sequencing. Twenty-four hours later the culture (10 mL) was used to prepare a glycerol bacterial stock inoculated into LB medium (200 mL, 37 °C, 225 rpm shaking) until the OD_600_ reached 0.4–0.6. After exposure on ice (25 °C), the cells were induced with IPTG with shaking (1 mM, 12 h, 25 °C), harvested by centrifugation (4000× *g*, in 4 × 50 mL tubes, 4 °C, 20 min), and the pellets stored (−80 °C).

### 2.4. hscFv Purification

For the hscFv purification procedure, the cell pellet (50 mL) from each culture (200 mL) was re-suspended in lysis buffer (3 mL, 20 mM Tris-HCl, 500 mM NaCl, 20 mM imidazole, 0.1% Triton X-100 pH 8.0). Cells were lysed on ice by sonication (6 × 30 s) and then centrifuged (14,000× *g*, 15 min) to remove cellular debris. The soluble fraction was filtered (0.2 μm filter) and applied to an HisTrap excel 1 mL column (Cytiva, Marlborough, MA, USA). The column was equilibrated and washed with 20 mM Tris-HCl, 500 mM NaCl, 20 mM imidazole, pH 8.0. A one-step purification elution of the sample was carried out at a constant flow of 1 mL/min (20 mM Tris-HCl, 500 mM NaCl, 500 mM imidazole, pH 8.0). The purified protein fractions (1 mL) collected through the elution were concentrated using a Millipore 10K Concentrator kept at 4 °C. Pooled fractions were resolved by size exclusion chromatography (25 mM Tris, pH 7.4, 150 mM NaCl, 0.02% NaN_3_). The purified protein was filtered (0.2 µm filter) and aliquoted in a biosafety cabinet. The purified protein was subsequently analyzed by SDS-PAGE, Western blot, SEC-UPLC, CE-SDS, and LAL methods.

### 2.5. Sodium Dodecyl Sulfate Polyacrylamide Gel Electrophoresis (SDS-PAGE) Analysis

The prepared scFv proteins were analyzed using SDS-PAGE. Reduced samples were mixed with sample loading dye (2×, 0.2% bromophenol blue, 4% SDS, 20% glycerol, 100 mM Tris-HCl, pH 6.8) and (100 mM dithiothreitol (DTT)). For non-reduced samples, DTT was excluded. After boiling (100 °C, 10 min), samples were loaded onto SDS-PAGE gels (12%). After electrophoresis, gels were stained (0.25% Coomassie brilliant blue, 50% ethanol, 10% acetic acid) and then destained (50% ethanol, 10% acetic acid), for imaging and visualization on the ChemiDoc XRS+ System (Bio-Rad, Hercules, CA, USA).

### 2.6. Western Blot of hscFv Fragments

The scFv proteins (20 µg) were separated by SDS-PAGE and blotted onto polyvinylidene difluoride (PVDF) membrane (7 min, iBlot2 Gel TransferDevice, Life Technologies, Carlsbad, USA) as we have described previously [3,4,5]. After the membranes were blocked (1 h, 5% *w/v* skimmed milk powder/TBS buffer, room temperature (RT)), the membranes were incubated with mouse anti-His antibody (1:5000, 1 h, GenScript, Piscataway, NJ, USA). After three washes (TBST buffer), the protein was visualized with HRP-conjugated donkey anti-mouse antibody (1 h, 3:10,000 in TBST, RT, Abcam, USA), followed by washing with TBS-T buffer (3X, 10 min). The protein was detected and visualized following the manufacturer’s recommendation (Amersham ECL detection reagent, Cytiva, Marlborough, MA, USA).

### 2.7. Protein Aggregation Analysis by Size-Exclusion Chromatography (SEC-UPLC)

Sample aggregation was assessed with ACQUITY UPLC Protein BEH size exclusion chromatography (SEC 200, 2 uL, 1 mg/mL, 1.7 μM, 4.6 × 150 mm column). The flow rate was 0.3 mL/min (10 min, mobile phase of 50 mM Sodium Phosphate, 500 mM NaCl, pH 6.2). Size-based separation was performed with UV detection.

### 2.8. Purity Analysis by CE-SDS

A high-throughput microchip capillary electrophoresis-based separation method was applied to analyze scFv purity (HT Protein Express assay, Microchip CE-SDS, PerkinElmer LabChip GXII Touch HT Protein Characterization System) [7]. Advantages of this advanced technology include accurate and quantifiable results, time efficiency, and minimal sample preparation artifacts. Samples (1 mg/mL) were run using default injection for both reduced and non-reduced separation conditions according to the manufacturer’s instructions. The CE-SDS data generated by LabChip were exported from each platform and imported into Empower^TM^ 3.8.1 Software (Waters Corporation, Milford, MA, USA) for analysis.

### 2.9. Octet RED384 Kinetic Measurements

Dilutions of the hscFv HC3-LC3 (5 μg/mL, 1X KB PBS pH 7.4, 0.02% Tween-20, 0.1% albumin, and 0.05% sodium azide) were dispensed into 384-well tilted-bottom microplates (90 μL/well). A second 384-well microplate was prepared containing human P2X4R peptide at 7 titrated concentrations in 3-fold serial dilutions (1.03–250 nM,) in glycine regeneration solution (pH 1.5) and 1X KB buffer for baseline stabilization. Both plates were continually agitated (1000 rpm). A total of 8 HIS1K penta-his antibody capture sensor tips per binding cycle were pre-hydrated (1X KB, 5 min, 8 sensors) before the binding measurements, followed by 3 pre-conditioning 15 sec dips in glycine (pH 1.5), alternated with 30 s dips in 1X KB. The sensor tips were then transferred to the scFv-containing wells for a 180 s loading step. After a baseline dip in 1X KB (30 s), the binding kinetics were measured by dipping the scFv-coated sensors into the wells containing varying concentrations of rat P2X4R peptide (GenScript). Binding interactions were monitored over a 240 s association period, followed by dissociation (420 s) in new wells containing fresh 1X KB buffer. All measurements were corrected for baseline drift subtracting a control sensor exposed only to running buffer. Data were analyzed using ForteBio data analysis software (Octet Analysis Studio software 12.2.0.20) (1:1 interaction model fitting global, Rmax unlinked by sensor).

### 2.10. Endotoxin Assay

Endotoxin levels were assessed following the manufacturer’s instructions (Toxin Sensor Chromogenic LAL Endotoxin Assay Kit, Cat. No. L00350, GenScript, USA), after diluting the HC3-LC3 hscFv with endotoxin-free water to adjust the concentration (1 μg/mL). The hscFv sample (100 μL. 1 μg/mL) or an endotoxin standard (1, 0.1, 0.05, 0.025, 0.01 EU/mL) was dispensed into an endotoxin-free vial. LAL reagent was added (100 μL), mixed, and incubated for 50 min (37 °C). After incubation, chromogenic substrate solution was added to each vial (100 μL), mixed, and incubated at 37 °C (6 min). Stop solution (500 μL, color-stabilizer #1) was added to each vial and mixed. Color-stabilizer #2 (500 μL), followed by color-stabilizer #3 (500 μL) was added to each vial and mixed (3 s). Absorbance was measured (545 nm,150 μL, each well of a 96-well plate) with a SpectraMax iD5 microplate reader (Molecular Devices, San Jose, CA, USA). Endotoxin units in a broad range (0.01–1 EU/mL) of standard solutions were calculated and plotted on a standard curve.

### 2.11. In Vivo Validation of hscFv Efficacy in FRICT-ION Chronic Trigeminal Neuropathic Pain Model

The Foramen Rotundum Inflammatory Constriction of the Trigeminal InfraOrbital Nerve (FRICT-ION) model of chronic facial pain was used to validate the efficacy of the HC3-LC3 lead. FRICT-ION is a minimally invasive, rapid method useful to induce facial allodynia for both rats and mice [8]. BALB/c white mice were primarily used through the 10 weeks of behavioral testing and digitally recorded behavior. Other coat colors and C57Blk6 have also be used with equivalent results in preliminary trials and in the spared nerve injury (SNI) model performed as an independent validation by Sai Life Sciences. In the anesthetized mice one lip was secured open with cotton suture to expose the buccal-cheek crease. A scalpel cut exposed the trigeminal nerve roots innervating the teeth and a 3 mm piece of chromic gut suture slid along the infraorbital branch of the trigeminal nerve into the foramen rotundum as it enters the skull. This surgery inside the mouth leaves no external evidence allowing study group blinding. The plateau phase neuropathic pain is considered chronic at 3 weeks when the single-dose hscFv treatment is given intraperitoneally (i.p.). The von Frey testing is performed weekly to validate continued hypersensitivity. Naïve mice remain untouched but undergo all behavioral testing. FRICT-ION produces hypersensitivity tested with von Frey filaments on the whisker pad, the innervation territory of the infraorbital nerve. The hypersensitivity develops over the subsequent 2 weeks and persists >100 days. Anxiety- and depression-like behaviors that develop in mice with nerve injury are tested after week 6. The P2X4R hscFv von Frey testing was performed in both male and female mice. Aligned with abundant literature for other P2X4 blocking treatments [2,9,10,11,12,13,14], female mice are resistant to the block of P2X4R, and thus other data for most tests beyond mechanical threshold testing is not provided for females.

### 2.12. Experimental Pain-Related Behavioral Read-Outs

Lead P2X4R hscFv HC3-LC3 with the best binding affinity was selected and tested for efficacy for reducing pain related measures in the FRICT-ION chronic neuropathic pain model, including the long-lasting hypersensitivity persisting > 100 days and development of anxiety- and depression-like behaviors after 6 weeks. A single injection of 4 mg/kg P2X4R hscFv or vehicle (phosphate buffered saline [PBS]) was given intraperitoneally (i.p.) to mice in week 2 or 4 post model induction to mice with FRICT-ION. Estimates are that in week 6, mice with these nerve injuries have experienced the equivalent of 8 years of chronic pain in humans [15], making this an ideal model for testing potential non-opioid therapeutics in vivo at time points relevant to patients with chronic pain.

#### 2.12.1. Reflexive Mechanical Threshold Using Von Frey Filaments

Mechanical hypersensitivity was tested at baseline and weekly thereafter with von Frey filament whiskerpad stimulation (5 applications, mid-range von Frey filaments) applied once every 3–4 s as we have reported previously [8,16]. Increased hypersensitivity was indicated by response to decreased gram force filaments.

#### 2.12.2. Cold Hypersensitivity Threshold

Cold hypersensitivity was tested once in weeks 6–8 by lightly pressing a cold copper coil (10 °C) on the whiskerpad.

#### 2.12.3. Cognitive-Dependent Anxiety- and Depression-like Behaviors Tested

Cognitive-dependent, non-reflexive behaviors were quantified once in week 8–10 after induction of the chronic model from video records collected in the absence of tester present in the room. Video-recorded behaviors were assessed by offline analysis.

a. Light/Dark Place Preference Test. Variables assessed in the two chamber anxiety-like behavior test were (1) time spent in each chamber, (2) number of transitions between chambers, (3) number of rearing events, and (4) entry latency into the light chamber [17,18]. 

b. Zero Maze Test. Anxiety/depression-like behavior was tested in the zero maze where a decrease in the distance and time traveled in the open portions of the maze is defined as a measure of anxiety/depression-like behavior [19,20]. Likewise, latency to enter the closed area is also measured during the 5 min test. 

#### 2.12.4. Power Calculation

a. von Frey Hypersensitivity. The power analysis provided the group size information based on the mean of the measures for the pilot in vivo assessment. Sufficient power for statistical significance was obtained using *n* = 3–6, using the methods described in our previous studies [8]. Group means were compared among experimental conditions by ANOVA. When appropriate, post hoc analysis was performed.

b. Anxiety/Depression. Anxiety/depression test latencies were analyzed by linear regression, using clustered standard errors to account for repeated measures and time as a covariate when appropriate.

In all cases, an α type-I error value of 0.05 is accepted for significant differences. All data are expressed as means +/− SEM.

## 3. Results

### 3.1. Humanization of P2X4R hscFv

**Figure 2:**
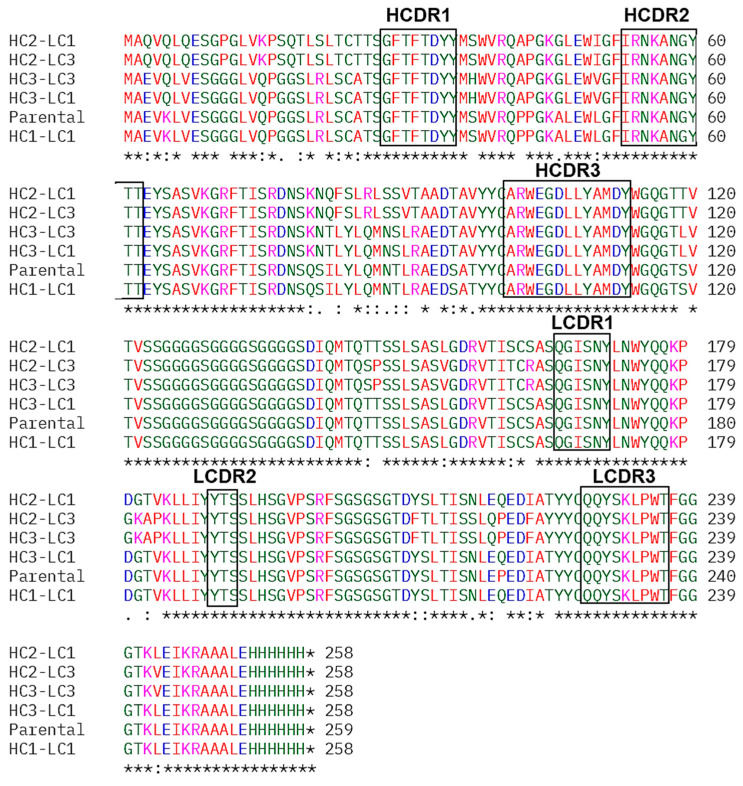
Amino acid sequences of humanized hscFvs. Amino acid sequences of five humanized hscFv candidates (HC1-LC1, HC2-LC1, HC2-LC3, HC3-LC3, and HC3-LC1) and their FRs and CDRs were determined (Clustal Omega, IMGT information system, respectively). A normal 15-amino-acid linker [(G4S)3] joins the HC and LC chains. Alignments were color-coded according to residue property groups. AVFPMILW—red, DE—blue, RK—magenta, STYHCNGQ—green, others—grey.

The most homologous human immunoglobulin germline V and J genes for FR donors to that of parental mscFv95 were searched based on sequence identity in Schrödinger’s BioLuminate. These identified human sequences served as the basis for grafting the antigen-binding regions (CDRs) from the mouse mscFv95. The three genes most homologous to the murine sequence were identified taking into account framework homology, maintenance of key framework residues, and canonical loop structure (based on a combined IMGT/Kabat CDR labelling approach) and were grafted to the human FR. The three CDR-grafted VH domains and three CDR-grafted VL domains were selected then to create and synthesize nine humanized scFv variants (3VH × 3VL) (Figure 2) that were cloned into the pET32a expression vector. Resultant recombinant chimeric scFvs were expressed and purified from the cytoplasm of *E. coli* Rosetta-gami cells, followed by assessment by ELISA and Octet for kinetic interaction analyses. Yields of up to 5 mg/L of pure, soluble, active hscFv fragments were achieved with a simple batch shake-flask culture.

Assessment of the humanness of the humanized hscFv variants by T20 Analyzer score of humanness ranged from 68.93 to 89.39 for the humanized full-length variable regions (HC and LC) and ranged from 78.3 to 96.9 for the humanized variable region frameworks. This exceeds the threshold of humanness. The humanness scores for the parental mouse and humanized antibodies are shown in Table 1. Humanization was successful with our method with each hscFv having a score of 80 or above, which is indicative of a human-like heavy-chain framework. A score of 90 or above, indicative of humanness for a kappa light-chain framework, was also achieved. For full-length variable regions, the recommended cutoffs of 79 for the VH and 86 for the VK were achieved [21].

### 3.2. Characteristics of the Lead HC3-LC3

#### 3.2.1. Specificity Binding by ELISA

**Figure 3:**
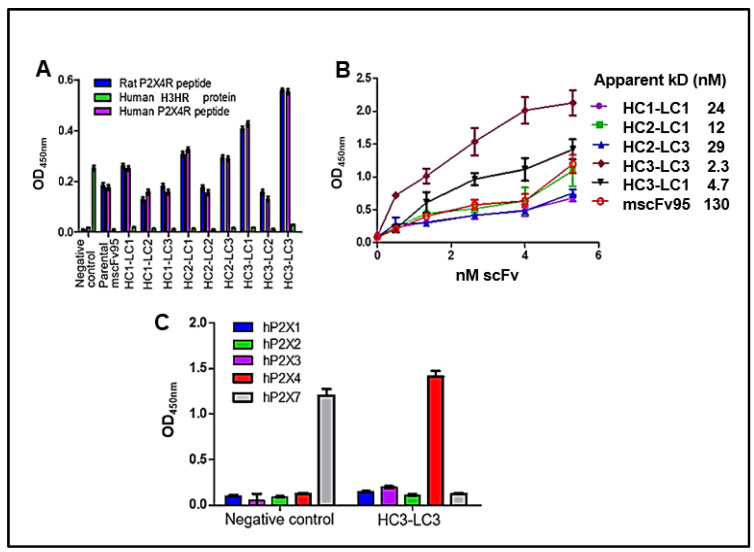
(**A**) Binding of scFvs to rat and human P2X4 peptides. The binding of the purified P2X4-humanized hscFvs in comparison to the parent murine scFv was tested by ELISA. The negative control was an scFv raised against histamine H3HR. (**B**) Binding of several of the purified humanized P2X4 hscFvs (HC1-LC1, HC2-LC1, HC2-LC3, HC3-LC3, and HC3-LC1) were compared and found to have better binding than the murine parent mscFv95. Each point represents the mean ± s.d. values of triplicate wells. (**C**) The hscFv with the best binding affinity, HC3-LC3, was selected as the lead. HC3-LC3 specifically bound to soluble human P2X4R but not to human P2X purinergic family receptors P2X1, P2X2, P2X3, P2X7. Anti-P2x7 bound only hP2x7peptide as a control.

Soluble, purified hscFvs were examined by ELISA assays (200 ng/well) to determine whether the humanized hscFvs could specifically bind to their corresponding rat and human P2X4 peptides. The humanized P2X4R hscFv bound only with rat and human P2X4 peptides, whereas the negative control humanized murine scFv 77-2 that binds histamine H3 has no cross-reactivity with human and rat P2X4 peptides (Figure 3A), although both P2X4 and histamine H3 are involved in pain [22].

The five promising hscFv clones (HC1-LC1, HC2-LC1, HC2-LC3, HC3-LC3, and HC3-LC1) identified from indirect ELISA bound to human P2X4 peptide with dissociation constants (KD) of 24, 12, 29, 2.3, and 4.7 nM, respectively, compared with the much higher KD of 130 nM for parental mscFv95 (Figure 3B). Among them, achieving more than a 10-fold affinity improvement, clones HC3-LC3, HC3-LC1, and HC2-LC1 exhibited the best binding affinity. Clone HC3-LC3 was selected as the lead for the in vivo validations with its over 50-fold enhancement compared with the parental mscFv95 sequence.

Further testing for specificity of binding to P2X4 found the lead HC3-LC3 hscFv bound only P2X4 and not the other purinergic P2X proteins tested (P2X1-P2X3, and P2X7). This provided better validity for the in vivo results (Figure 3C).

#### 3.2.2. Gel Electrophoresis and Western Blot

**Figure 4:**
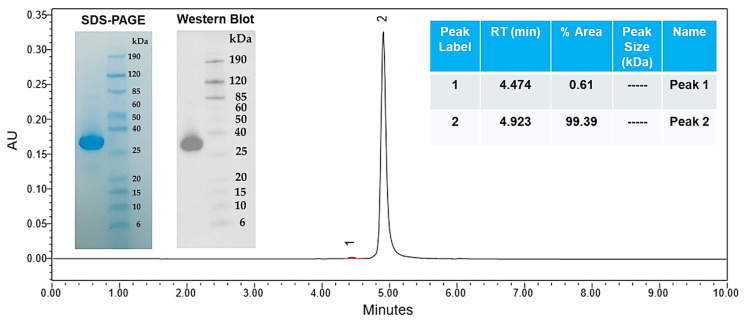
SDS-PAGE and Western blot analysis. SDS-PAGE and Western blot analysis of HC3-LC3 hscFv were run under reducing conditions post SEC-UPLC. SEC-UPLC exhibits > 95% purity.

**Figure 5:**
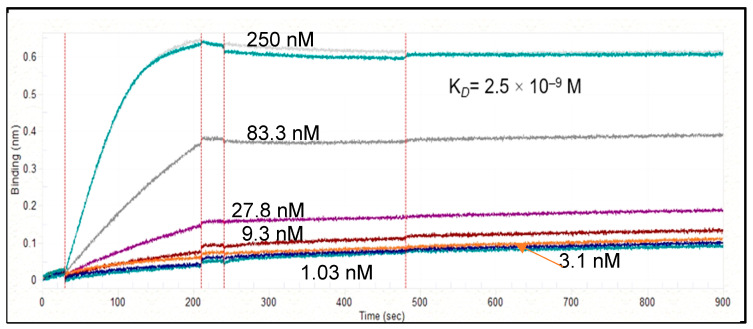
HC3-LC3 and human P2X4R peptide affinity determination on the Octet RED384 instrument of three-fold serial dilutions.

**Figure 6:**
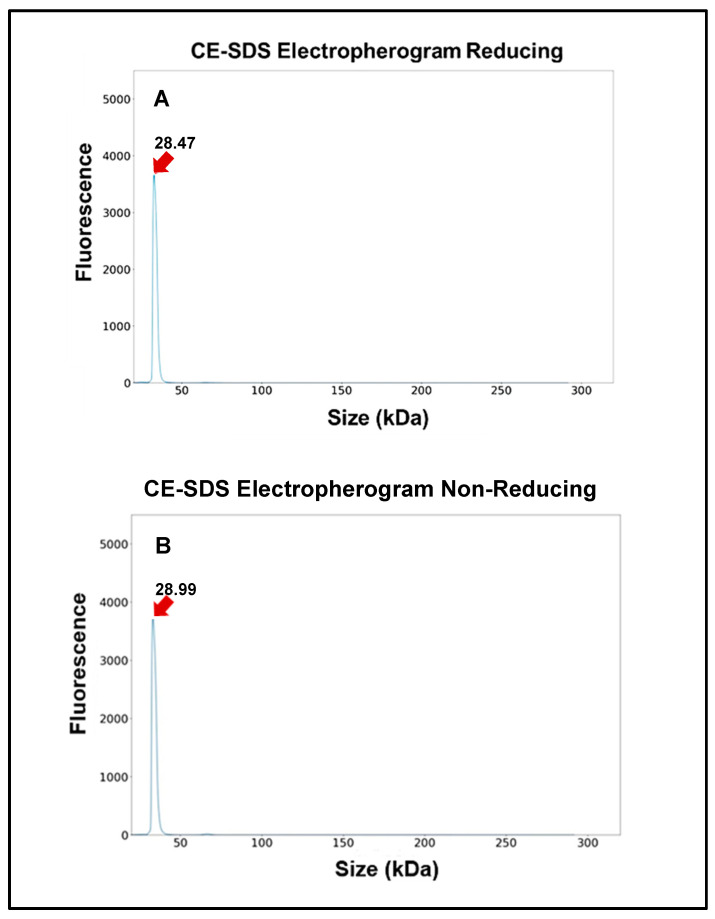
Electropherograms for P2X4 hscFv are shown both from reduced (**A**) and non-reduced (**B**) CE-SDS.

The HC3-LC3 exhibited a single band with an apparent molecular mass 28 kDa, which is within the expected size for the monomeric form of this protein. This was determined in gel electrophoresis and Western blot (Figure 4). Octet measurements further revealed that the HC3-LC3 had binding kinetics of KD = 2.5 × 10–9 M (Figure 5). This hscFv molecule also maintained the required monomer resolution on size exclusion chromatography (SEC-UPLC) and capillary electrophoresis (CE-SDS) under denaturing and reducing conditions (Figure 6A,B). The lead HC3-LC3 clone was selected for further testing in in vivo chronic pain models.

#### 3.2.3. Endotoxin Content

**Figure 7:**
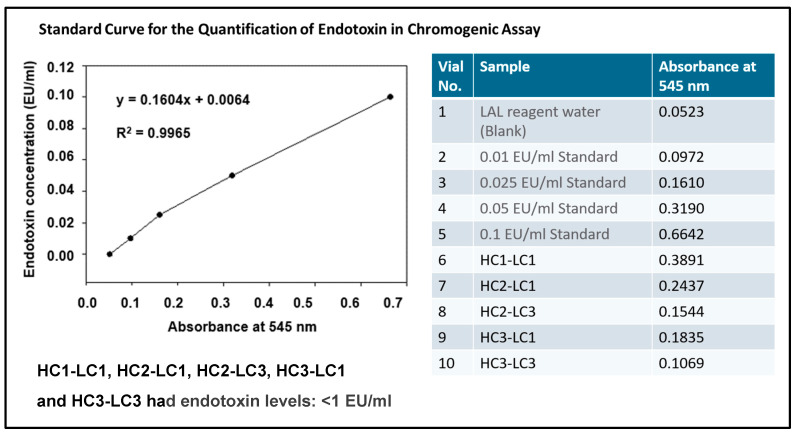
Endotoxin concentrations were assessed under standard conditions. Absorbance at 545 nm was determined against the blank and four standards plotted in a best-fit linear relationship in the concentration range of 0.01–0.1 EU/mL. The table provides the absorbances for the standards and five hscFvs.

As required for use, the endotoxin level in this protein was < 1.0 EU/mL as determined by the chromogenic LAL method, which provides accurate in vitro end product endotoxin detection. The assay was run under standard conditions with absorbance at 545 nm showing a linear relationship (Figure 7). The HC1-LC1, HC2-LC1, HC2-LC3, HC3-LC1, and HC3-LC3 hscFv all showed minimal endotoxin levels: < 1 EU/mL, with the lead HC3-LC3 reading 0.1069 EU/mL.

#### 3.2.4. pK for Humanized P2X4 hscFv Biologic

**Figure 8:**
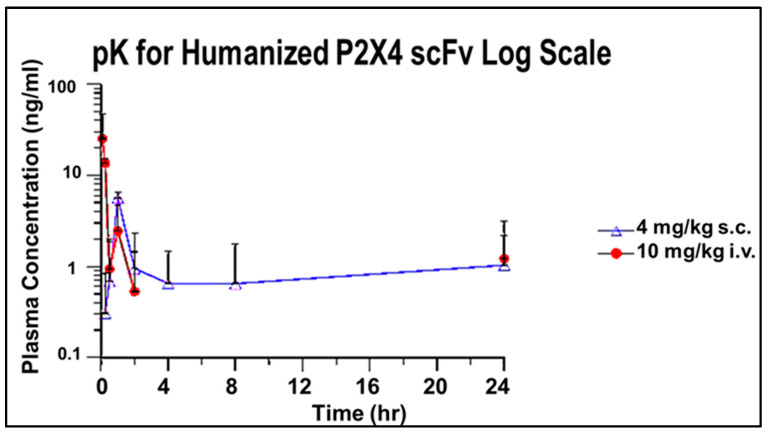
P2X4 hscFv is measurable in the plasma for over 24 h. Plasma concentration was measureable for over 24 h and was determined for both the 4 and 10 mg/kg doses.

Typically, scFvs are shown to persist for only 3–4 h due to clearance by the kidney. Figure 8 is a chromatogram of plasma from mice treated with the P2X4 hscFv generated by a commercial vendor from the patent sequence. Measurable levels of the hscFv were found in plasma for over 24 h when administered either subcutaneously (s.c., 4 mg/kg) or intravenously (i.v., 10 mg/kg) (Figure 8). The persistence of the P2X4 hscFv after administration indicates the hscFv is widely distributed about the body for over 24 h.

### 3.3. Validation with Reversal of Nerve Injury Chronic Neuropathic Pain by HC3-LC3 hscFv

Optimal dose of the humanized hscFv lead (best affinity binding) produced significant reversal of pain-related behaviors as in the previous study with the parent murine mscFv, i.e., 4 mg/kg. The HC3-LC3 hscFv was tested for efficacy in reduction of trigeminal nerve FRICT-ION and SNI chronic neuropathic pain models. The hscFv was given either in week 2 or 4 after model induction to observe efficacy when given at different time points after injury. The persistence of the chronic trigeminal pain model allowed cold testing and spontaneous emotive responses once each in weeks 6–8.

#### 3.3.1. Von Frey Mechanical Hypersensitivity

**Figure 9:**
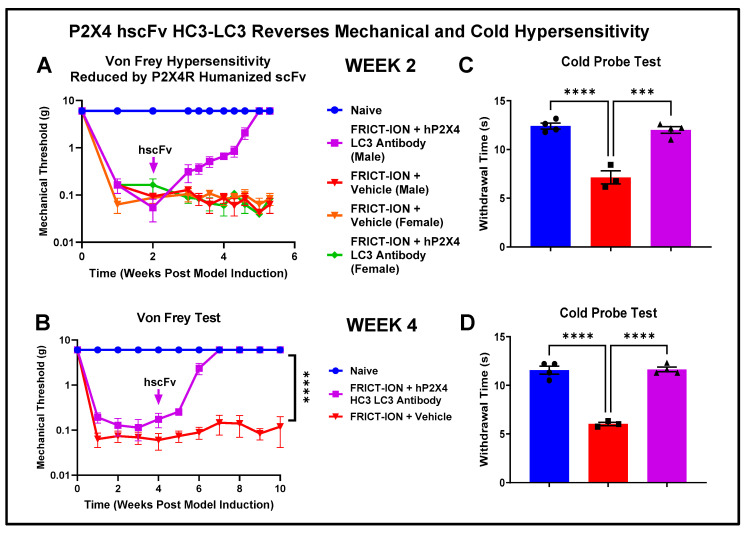
Single-dose humanized hP2X4R HC3-LC3 hscFv effectively reverses mechanical and cold hypersensitivity in male but not female mice (**A**). (**A**) Single 4 mg/kg dose of the P2X4 hscFv was given in week 2, reversing the von Frey threshold to naïve baseline in male mice but not female mice (*n* = 6). (**B**) Treatment in week 4 after induction of the FRICT-ION trigeminal chronic neuropathic pain model was also effective in reversing the mechanical threshold to naïve baseline with the single dose. The von Frey testing was continued once each week demonstrating the durability of single dose of P2X4R hscFv HC3-LC3 (4 mg/kg) through >10 weeks (*n* = 4). Two-way ANOVA (Dunnett’s multiple comparison’s test), **** *p* < 0.0001) (**C**). Cold hypersensitivity tested in week 1 was reversed in the mice with FRICT-ION when P2X4 hscFv was given in week 2 (*n* = 4). (**D**) Cold hypersensitivity is also reversed in the mice with FRICT-ION when P2X4 hscFv was given in week 4 (*n* = 4). *** *p* < 0.001 vs. FRICT-ION, 2-way ANOVA; **** *p* < 0.0001 vs. naïve or FRICT-ION.

The FRICT-ION trigeminal nerve chronic neuropathic pain model develops hypersensitivity over the subsequent 2–3 weeks and persists for at least 100 days in BALBc mice. The FRICT-ION model produces a durable hypersensitivity response persisting 5–7 weeks after model induction. The humanized P2X4R hscFv lead, HC3-LC3, was given as a single dose (4 mg/kg) either in week 2 (Figure 9A) or week 4 (Figure 9B) after induction of the FRICT-ION nerve injury model. The HC3-LC3 hscFv provided successful reversal of mechanical hypersensitivity to naïve baseline. The prolonged recovery time course was similar to that of the parent murine mscFv we reported previously [2].

#### 3.3.2. Reversal of Cold Hypersensitivity

In mice with FRICT-ION that received hscFv HC3-LC3, there is no sensitivity to a cold copper coil (10 °C) applied to the ipsilateral face. The cold coil response was similar to that of naïve mice and was significantly different for untreated mice with FRICT-ION (Figure 9C,D). Cold is a particularly noxious stimulus for patients with craniofacial pain.

#### 3.3.3. Relief of Anxiety- and Depression-like Behaviors

**Figure 10:**
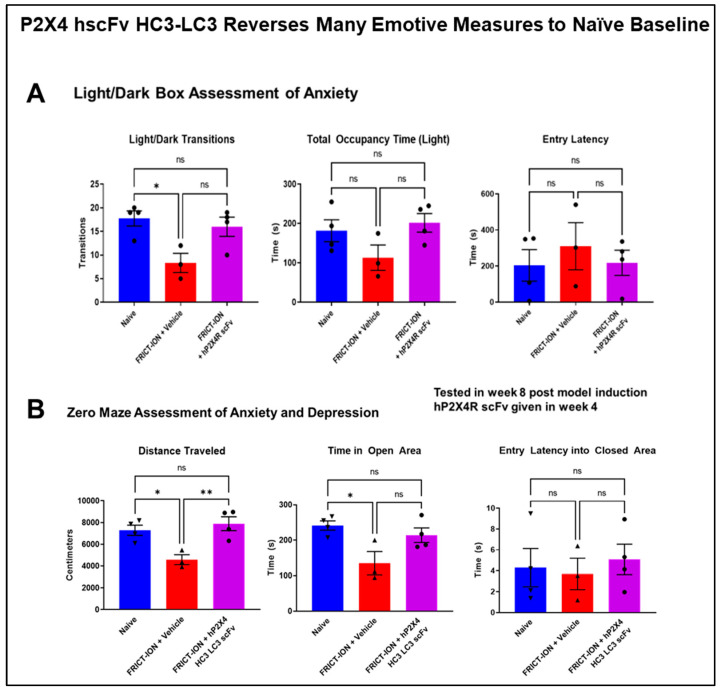
(**A**) Anxiety-like measures with the light/dark place preference test indicated are altered in mice with FRICT-ION chronic neuropathic pain. P2X4R hscFv prevented development of the anxiety-like behaviors observed, including number of transitions between the boxes, time spent in the light, and decreased latency for lighted box entry which are similar to naïve controls. Data is shown for the mice treated in week 4. *n* = 4, one-way ANOVA (Dunnett’s multiple comparisons test); * *p* < 0.05, (**B**). FRICT-ION model alters most measures observed with the zero maze test, including distance traveled, time in the open areas, and latency to first entry into the closed area. Anxiety/depression behaviors with P2X4R hscFv-treated mice are similar to naïve control. Data is shown for the mice treated in week 4. *n* = 4, ns = not significant, one-way ANOVA (Dunnett’s multiple comparisons test); * *p* < 0.05, ** *p* < 0.01.

Testing after 6–8 weeks in mice given P2X4 hscFv found reduced anxiety/depression-like behaviors comorbid in the absence of the chronic pain. Several measures were reversed testing anxiety with the light/dark box test in mice with the humanized hscFv treatments (Figure 10A). The zero maze anxiety/depression measures in mice treated with the P2X4 hscFv were also significantly different from untreated mice but not significantly different from the control mice (Figure 10B). Reversal of the chronic hypersensitivity back to naïve baseline in the prior weeks provided by the P2X4 hscFv prevented the development of the anxiety- and depression-like behaviors typically seen in the chronic model in weeks 6–8 post model induction.

### 3.4. Validation with Reversal of Spared Nerve Injury (SNI) Chronic Neuropathic Pain by P2X4 hscFv

**Figure 11:**
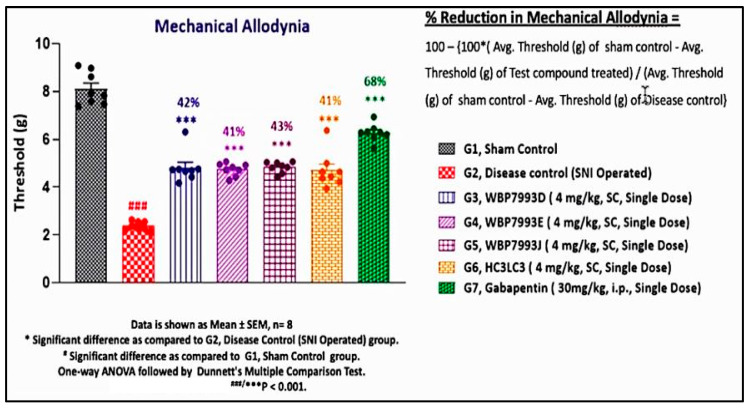
Effectiveness of P2X4 hscFv protein was generated from the patent sequence. C57Blk6 male mice were subjected to the spared nerve injury (SNI) model and after 3 weeks given a single subcutaneous dose of the hscFv generated by the commercial vendor (4 mg/kg) for comparison to sham controls. The footpad was tested 4 weeks after treatment with a Dynamic Plantar Aenesthesiometer (Ugo Basile, Gemonio VA, Italy, max force 20 g at 10 s of ramp). Percent reduction in mechanical allodynia was between 41 and 43% at 4 weeks after treatment with a single subcutaneous dose of the hscFv. In comparison, gabapentin reduces mechanical allodynia 63% (single dose, 3 h), but the effect of gabapentin is not durable.

Replicates of the P2X4 hscFv were generated from the published patent sequence under a contractual agreement with a commercial vendor (Wuxi AppTec, Shanghai, China). The functional efficacy of the four replicates was independently tested by another contractor (Sai Life Sciences, Telengana, India) with the spared nerve injury model in C57/Blk6 mice (Figure 11). The mice were given a single dose of hscFv three weeks after the SNI was induced. Four weeks later the mice were tested for von Frey mechanical hypersensitivity on the footpad in a comparison with gabapentin, the standard of care. The durable increase in the threshold provided by the P2X4 hscFv replicate four weeks after treatment was compared to the acute treatment of gabapentin at 3 h (Figure 11).

## 4. Discussion

### 4.1. Comparison of Ribosome Display-Generated hscFv Biologic to Other Immunotherapies

Among current clinical antibody-based therapies and immune modulators, numerous full-length antibodies are currently in use that are designed to reduce immune factors or migraine. Monoclonal antibodies are produced traditionally through rodent immunization using hybridoma technology, such as the CGRP monoclonals for migraine. However, this approach is laborious, expensive, and poses difficulties, including generating antibodies against self-antigens.

Numerous scFv-based therapies and immune modulators are currently in use, particularly for cancer therapies since they are so effective in engaging specific targets. This more efficient and cost-effective scFv technology has already been applied to target proteins known to be linked to central nervous system neurodegenerative diseases. Immunotherapies, including scFvs, are available for a diversity of neurological disorder targets such as semaphorin 4D (Huntington’s disease); nerve growth factor; tau; α-synuclein; and amyloid β (Alzheimer’s disease, ALS, Parkinson’s disease) [23,24]. The literature acknowledges that scFv technology is a promising alternative to full-length immunoglobulins. For the nervous system it offers important advantages compared with conventional monoclonal antibodies. Advantages include extreme specificity, brain penetrance, superior stability and solubility, higher affinity, reduced self-immunogenicity, as well as easier and less expensive large-scale production.

Therapy based on the small scFv format overcomes the previous challenges of providing therapeutic applications for P2X and other receptors, which have been problematic [25,26,27,28]. Smaller engineered antibodies, such as scFvs, feature similar binding activity but stronger tissue and brain penetrability according to previous studies. These qualities make scFv antibodies well suited for selective targeting of P2X4 and further development as chronic pain therapy, particularly since P2X4 is only present in low levels under normal conditions but greatly increases after nerve injury [28].

In recent years, in vitro phage and ribosome display techniques have become platform technology for the design, selection, and production of reagents for targeted therapies, including for P2X3 [29]. The in vitro ribosome display technology we use has a number of advantages over phage display technology, and it is used widely to select scFvs against a variety of targets [3,4,5,30,31,32,33,34].

### 4.2. Advantages of Utilizing the Specific Ribosome Display and Humanization Technology Used Here to Generate the P2X4 hscFv

The small scFv antibodies targeting P2X4, when humanized, remained in the size range that would not generate an immune response in a human host, and effort is underway to determine if they cross the blood/brain barrier as we have seen for the parent murine P2X4 mscFv with Western blots [2]. The selectivity demonstrated for binding to P2X4 and not the other purinergic receptors tested indicates their importance as potential specific therapy that could slow the process they initiate related to pain and inflammatory cascades. Though not immediate, the effectiveness of the P2X4 hscFv provided by treatment in weeks 2 or 4 post injury allowed the hypersensitivity processes to subside only in the treated mice over time. The reversal of pain measures over two weeks’ time is an interesting phenomenon requiring much additional study.

Our study generated a panel of humanized *P2X4*-specific single-chain (scFv) antibodies superior to that of our previously identified lead murine parent mscFvs. The persistence of the hscFv in the blood beyond 24 h demonstrated here is an unusual feature not common among other scFvs, implying delayed clearance. The parent mscFv was generated using cell free ribosome display technology, with PCR products cloned into pGEM-T vectors to transform *E. coli*-competent cell-based upscaling. Although humanization can sometimes restrict binding to murine sequences, the selected hscFv lead, affinity binding of the lead hscFv was improved into the nanomolar range compared to the parent mscFv, and the low endotoxin level was acceptable. This was likely due to the rapid single-step purification. We used affinity chromatography for the initial antibody capture, followed by SEC as a polishing step.

The lead humanized hscFv effectively provided reversal of the pain-related behaviors, returning them to baseline within 2–3 weeks following a single administration (4 mg/kg, i.p.). Non-reflexive measures of anxiety and depression that typically develop in untreated mice within 6–8 weeks in chronic pain models were decreased or did not develop in the absence of chronic pain. Evaluations of behavioral measures in the chronic trigeminal nerve injury neuropathic pain model indicate durable effectiveness of P2X4 hscFv as a potential pain therapy. Effectiveness for pain behaviors is not immediate, but the hscFv binds to the P2X4R sequence selected, not the P2X4 activation site. Clearly, the binding to the selected peptide sequence contributed to the winding down of the hypersensitivity by some as yet unknown means through other downstream processes. This is an avenue we are pursuing to determine the mechanism(s) of action.

### 4.3. Significance in the Context of Prior Research

Purinergic P2X1-7 channels have been identified as important drug targets since it was noted that ATP injection into the skin of human volunteers caused pain and subsequently because of their involvement in pain following nerve damage and associated inflammation [25]. Neuropathic and acute inflammatory pain and other responses in female animals remain unchanged when P2X4 hscFv is given in our study. As noted from the earliest reports to the present, there is sexual dimorphism for P2X4 activation [10,11,26]. The data at chronic time points extends this well-known report that block of P2X4R is ineffective in providing relief of pain-related behaviors in female mice. Nonetheless, we selected a P2X4 sequence as promising a therapeutic target since it was unique from that site in other purinergic receptors.

The importance that P2X4R are upregulated after nerve injury and play a crucial role in chronic and inflammatory pain signaling has been the point of many previous pursuits to prevent its initiation of the cascades evoking neuropathic pain [35,36]. P2X4, an ATP-gated nonselective cation channel highly permeable to calcium, is primarily localized on dorsal root ganglia (DRG), satellite glial cells, and spinal cord microglial cell membranes [27,37,38,39,40]. Injury-induced activation of P2X4 also causes inflammatory mediator release from satellite glial cells [41,42]. Increased primary afferent firing after injury activates spinal dorsal microglial release of pro-inflammatory cytokines TNF-α, IL-1β, IL-6, ATP, NGF, NO, ROS, PGs, and other active neuronal substances that participate in maintenance of neuropathic pain [41]. Transfer of ATP-primed macrophages into the paw provokes hyperalgesic responses in naïve mice but not in P2X4-deficient mice [27]. In a rat model of neuropathic pain, Tsuda et al. [28] found that intraspinal antisense knockdown of P2X4 decreased cold hypersensitivity just as it did in wild-type mice. Numerous pain model studies have shown hypersensitivity does not develop in P2X4 knockout mice [28,42]. Furthermore, the anti-allodynic effects of a selective P2X4 antagonist, NP-1815-PX, supports the hypothesis that microglial P2X4 is a potential target for treating chronic pain [43]. Most previous studies have been performed in acute pain models, and it was not known if longer-term block of P2X4 receptors would significantly reduce neuropathic pain. This study addressed these important knowledge gaps.

### 4.4. Strengths and Innovation of the scFv Biologic for Chronic Pain

We described here the unique ribosome display platform we developed and used to generate hscFvs based on eukaryotic ribosome display technology [3,4,44,45]. The ribosome display platform is a superior in vitro cell-free platform that generates peptide, protein, and antibody libraries. The products have unparalleled diversity up to 10^13–15^ and no transformation is required. As a result, extremely high-affinity binders can be isolated from the libraries against a variety of targets. Our ribosome display antibody libraries produce antibodies with nanoM/picoM affinity, the highest affinity ever achieved by an antibody production technology. Additional mutations can also be introduced into the gene by PCR.

Another innovation of this project includes the success of the P2X4-specific humanized hscFv therapy for the first time in in vivo testing in the mouse *chronic* neuropathic pain model. The success of the project reiterates the value of scFvs for future clinical studies and serves as a proof of concept for successful development of non-opioid therapeutic interventions for chronic pain.

### 4.5. Limitations of the Study

The mechanism of action for the P2X4 hscFv is unknown at this time. Binding of the hscFv to native P2X4 other than in mice has not yet been tested, so it is not known how binding of hscFv to P2X4 modulates its function ex vivo. Since the hscFv was designed to bind to an extracellular peptide sequence, not the activation binding site, we believe this is a key factor explaining both the efficacy and the delayed action, but this must be examined in the future. Neither is it known if the hscFv is crossing the blood/brain barrier due to the small size, as has been demonstrated with Western blot for the murine parent scFv [2]. Liu et al. (2019) demonstrated that their much larger scFv and conjugated scFvs (50 and 120 kD, i.v.) crossed into the brain using IVIS, MRI imaging, and the in vitro BBB transwell system [46]. Other mechanisms have been presented in the literature, such as transcytosis or nonspecifically simply as a cell-penetrating peptide. A review is cited here for descriptions of these scFv mechanisms for crossing the cell membrane [47]. Effectiveness for the reversal in male mice with the chronic nerve injury-induced pain model partially validates use of P2X4R hscFv as effective therapy for chronic nerve injury-induced neuropathic pain. Other equivalent pain relief therapies for women must be developed among current dimorphic receptor actions being discovered.

## 5. Conclusions

Our laboratories have refined a rapid eukaryotic ribosome display method to develop panels to target P2X4 [2] and cholecystokinin B (CCK-B) receptors [5] to reduce hypersensitivity, anxiety, and depression in our chronic pain models. The method is inexpensive, rapid, and can be used to quickly develop high-affinity antibodies that can be readily modified to incorporate intrinsic fluorescence for in vivo imaging in an animal model. The scFv biologics are known to cross the blood/brain barrier due to their small size and water solubility. In the case of this hscFv binding P2X4, receptor-mediated transcytosis or straightforward cell penetration of the protein may be the mechanism of action [33] for the effectiveness in altering this well-known initiator of central and peripheral pain signaling pathways. The unusual retained presence in the plasma may also be a contributor to its effectiveness. Here we applied ribosome display technology to develop a panel of novel therapeutic small molecules against a fragment of P2X4R unique from other P2Xs. Large libraries obtained in triplicate provided sufficient material to screen for high-affinity hscFvs and validate the results. P2X4 is abundant in nervous tissue, but it is inactive remaining sequestered in endosomes unless intense nerve activation or tissue damage occurs. Administration of the humanized P2X4 scFv was well tolerated and effectively rescued both trigeminal and sciatic neuropathic pain models. Binding specificity for P2X4 and not several other P2X proteins (P2X1-P2X3, and P2X7) assures validity of the results presented. The nature of the significant delay in restoration of naïve baselines in all the measures is as yet not known.

The scFvs are providing new therapeutics, overcoming previous challenges of providing therapeutic applications for neuronal, pharmacology, and pathophysiology research applications [48]. Due to their solubility and small size, scFvs are being investigated and utilized as therapeutics for nervous system conditions such as arthritis and Creutzfeldt–Jacob and Huntington’s diseases [49,50,51,52]. These brain-penetrant molecules have many promising biotherapeutic applications for treatment of both the nervous and immune systems, which are known to be interactive in chronic pain [50,51].

Opioids are limited by their serious side effects, including sedation, respiratory depression, constipation, tolerance, and opioid dependence. Therefore, non-opioid therapeutics with higher efficacy and fewer side effects, such as P2X4 hscFv, for chronic pain are urgently needed. Current understanding of the widespread pathophysiological functions of purinergic receptors has indicated the importance of specific therapeutic development targeting not only chronic pain but the many other clinical syndromes impacted by purinergic co-transmission and cellular signaling, as recently reviewed by the originator of this idea, Geoffrey Burnstock [53].

## 6. Patents

These studies are protected under the following US patents:

U.S. Patent No. 12,145,991, “Therapeutic Antibody Fragments, Methods of Making, and Methods of Use” Authorized by Karin Westlund High, Ravi Venkata Durvasula, Adinarayana Kunamneni. Albuquerque, NM (US)Published 13 November 2024.

International Patent No. WO 2023/114962 A1, “Humanized Non-Opioid Composition and Therapies for Pain Management-P2X4 ScFv”. Authorized by Karin Westlund High, Adinarayana Kunamneni, and Sascha Alles, Published 22 June 2023.

US Patent File # WO 2023/114962 A1 Humanized Non-Opioid Composition and Therapies for Pain Management-P2X4 scFv. Karin Westlund High, Adinarayana Kunamneni, Published 22 June 2023.

US Patent No. 2022/0298238 A1, “NON-OPIOID COMPOSITIONS AND THERAPIES FOR PAIN MANAGEMENT”. Authorized by Karin Westlund High, Ravi Venkata Durvasula, Adinarayana Kunamneni. Albuquerque, NM (US). Filed 23 August 2019, Published 22 September 2022.

US Patent File #0310.000163US60. Sascha R.A. Alles and Karin Westlund High (2020) Compositions and methods for alleviating pain. Published 5 May 2022.

US Patent Application Pub. No. US 2021/0340265 A1, Therapeutic Antibody Fragments, Methods of Making, and Methods of Use. Authorized by Karin Westlund High, Ravi Venkata Durvasula, Adinarayana Kunamneni. Application No. 17/284,208, filed 9 April 2021. Published 4 November 2021.

## Figures and Tables

**Table 1:** Humanness assessment for humanized and mouse P2X4R scFv95.

Variable Region Chain	Species	T20 Analyzer Score (Full Length)	T20 Analyzer Score (Framework Only)
mscFv95-HC_parental	Mus musculus	74.5	78.3
hscFv95.HC1	Homo sapiens	74.51	78.33
hscFv95.HC2	Homo sapiens	68.93	84.88
hscFv95.HC3	Homo sapiens	82.09	90.92
mscFv95-LC_parental	Mus musculus	76.9	82.3
hscFv95.LC1	Homo sapiens	76	81.06
hscFv95.LC2	Homo sapiens	88.7	96.9
hscFv95.LC3	Homo sapiens	89.39	96.88

## Data Availability

The raw data supporting the conclusions of this article will be made available by the authors on request.

## Abbreviations

The following abbreviations are used in this manuscript:

scFv	Single-chain fragment variable
VH	Variable heavy chain
VL	Variable light chain
CDR	Complementary determining region
hscFv	Humanized single-chain fragment variable
P2X4R	P2X purinoceptor 4
FRICT-ION	The Foramen Rotundum Inflammatory Constriction of the Trigeminal InfraOrbital Nerve
SNI	Spared nerve injury
*CGRP*	*Calcitonin gene-related peptide*
KD	Dissociation constant
PCR	Polymerase chain reaction
ELISA	Enzyme-linked immunosorbent assay
SDS-PAGE	Sodium dodecyl sulfate/polyacrylamide gel electrophoresis
SEC-UPLC	Size-exclusion ultraperformance liquid chromatography
LAL	Limulus amebocyte lysate
IL-6	Interleukin-6
CD-54	Cluster of differentiation 54
ANOVA	Analysis of variance
